# Sex, sex chromosomes and gene expression

**DOI:** 10.1186/1741-7007-9-30

**Published:** 2011-05-04

**Authors:** Xuemei Lu, Chung-I Wu

**Affiliations:** 1 Laboratory of Disease Genomics and Individualized Medicine, Beijing Institute of Genomics, Chinese Academy of Sciences, Beichen West Road, Chaoyang, Beijing, China; 2 Department of Ecology and Evolution, University of Chicago, 5801 South Ellis Avenue, Chicago, IL 60637, USA

## Abstract

The X chromosome has fewer testis-specific genes than autosomes in many species. This bias is commonly attributed to X inactivation in spermatogenesis but a recent paper in *BMC Biology *provides evidence against X inactivation in *Drosophila *and proposes that somatic tissue- and testis- but not ovary-specific genes tend not to be located on the X chromosome. Here, we discuss possible mechanisms underlying this bias, including sexual antagonism and dosage compensation.

See research article {http://www.biomedcentral.com/1741-7007/9/29}

## 

In species with chromosomal sex determination, genic contents might reasonably be expected to be different among the two sex chromosomes and the autosomes. After all, the X chromosome spends two-thirds of the time in females, autosomes one-half and Y none at all, leading to differing selective pressures on X-linked genes between the sexes. The theory of sex-dependent selection would predict that, relative to the autosomes, the X is expected to be moderately female-biased in gene content and Y to be extremely male-biased [1,2]. There is evidence for this expected feminization of the X chromosome [1,2], manifested in the localization of female-biased genes on the X and male-biased genes on the autosomes or Y.

## Meiotic sex chromosome inactivation driving biased gene localization

**Figure 1:**
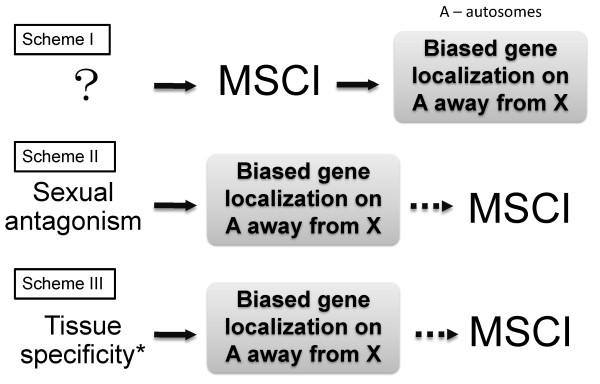
**Three evolutionary schemes to explain gene localization between X and autosomes (denoted as A)**. The grey box indicates the preferential localization of male-biased (including testis-specific) genes on the autosomes and away from the X chromosome. In each scheme, a different force drives this biased localization. The dotted arrow indicates a step that may or may not follow. Scheme I: MSCI precedes and drives biased gene localization through selective pressure against X-linked spermatogenesis genes, which would fail to be properly expressed in spermatocytes. Scheme II: sexual antagonism leads to biased gene localization through selective pressure for X-linked female-advantageous but male-disadvantageous mutations (and against the opposite). If this leads to all spermatogenetic genes on the X chromosome being selected against, conditions are right for MSCI to possibly arise. Scheme III: improper regulation of tissue-specific genes on the X chromosome leads to biased gene localization through selective pressure against tissue-specific genes on the X chromosome. We suggest that a plausible mechanism for the X/A dependence of tissue-specific expression reported by Mikhaylova and Nurminsky may be dosage compensation (indicated by an asterisk). Again, this leads to conditions in which it is possible for MSCI to arise.

Despite the theoretical prediction of biased gene localization on sex chromosomes, the most widely cited explanation for this bias is not a theoretical one but an empirical observation commonly referred to as meiotic sex chromosome inactivation (MSCI). MSCI is easily observable in mammals by cytogenetic means [3]. In mature sperm, all chromosomes are inactivated but it is the X chromosome that is inactivated first. MSCI is somewhat counter-intuitive as it leads to inactivation of the only X chromosome in XY males whilst maintaining expression from both copies in XX females. Since the X chromosome is inactivated precociously in spermatocytes, genes required for sperm maturation would be expected to be on (or escape to) the autosomes. This is illustrated as Scheme I in Figure 1[4,5]. In this scheme, MSCI is the driving force of the biased gene localization. In a new study in *BMC Biology*, Mikhaylova and Nurminsky suggest that this standard explanation may not be applicable, at least in *Drosophila *[6].

Despite the wide acceptance of Scheme I, it has been reported to have major weaknesses [2,7]. First, the driving force behind MSCI has not been clearly identified. There are a number of suggested mechanisms [8] but most seem *ad hoc *given the drastic action spermatocytes commit themselves to. Second, if gene localization is driven by MSCI, then genes pertaining to sexual characteristics not directly related to gametogenesis would not be expected to show bias in chromosomal localization. Nevertheless, X-linked genes appear to avoid male-biased expression in non-gametogenic tissues [1] such as accessory glands. Third, evolution rarely progresses by fitness loss (MSCI) followed by fitness gain (gene re-localization). In that sense, the scheme follows a path rarely travelled.

Mikhaylova and Nurminsky [6] carried out a study that appears to negate the existence of MSCI in *Drosophila*. Using microarray analysis and quantitative RT-PCR to track the development of the testis, they observed no reduction in the expression of X-linked genes as sperm mature. While acknowledging the genetic evidence for precocious X inactivation [3,7], the authors interpret the observed expression pattern to be inconsistent with the predictions of MSCI. The eventual resolution of this apparent contradiction may go either way. Microarray and quantitative RT-PCR data reveal the abundance of transcripts, but not the rate of transcription. If transcripts in the testis are unusually stable due to the extensive post-transcriptional regulation of spermiogenesis, then the microarray data may not reveal reduction in X-linked transcription during spermatogenesis. On the other hand, as Mikhaylova and Nurminsky point out, the genetic evidence for precocious X inactivation in *Drosophila *is indirect [6].

## Other possible forces driving biased gene localization

If MSCI is indeed absent in *Drosophila*, an alternative scheme must be sought to account for the bias in the localization of tissue-specific genes to particular chromosomes. The sexual antagonism driving the X inactivation (SAXI) hypothesis may be such a scheme (Scheme II of Figure 1) [7]. In this hypothesis, chromosomal localization of genes is driven by sexual antagonism under which, because the X chromosome spends more time in females than males, X-linked mutations that benefit females at the expense of males are more likely to be fixed than autosomal mutations that do the same [9]. SAXI, it is suggested, drives X to be female-biased and autosomes to be slightly male-biased, relative to the X. If mutations in genes involved in late spermatogenesis are more likely to be sexually antagonistic than those in other genes, the X will eventually become dispensable for late spermatogenesis [7]. Therefore, in Scheme II, SAXI makes it possible for MSCI to evolve, but does not predict its evolution. Indeed, in a recent study of X:autosome imbalance driving male sterility in *Drosophila*, Lu *et al*. [10] used the framework of SAXI without requiring MSCI.

In their report, Mikhaylova and Nurminsky suggest a new and quite unexpected explanation, shown in Scheme III of Figure 1. They observed that all genes showing tissue-specific expression, including in the testis and somatic tissues such as the midgut, are under-represented on the X chromosome. The prominent exception to this is genes expressed specifically in the ovary. They suggest that the low number of testis-specific genes present on the X chromosome is likely to be driven by the same mechanism as the low number of other tissue-specific genes and is therefore unlikely to reflect either MSCI or SAXI. Instead, they suggest that there is a lack of efficient tissue-specific gene regulation on the X chromosome, creating selective pressure for genes requiring such regulation to relocate to autosomes.

To account for the autosomal bias in non-reproductive tissues, Mikhaylova and Nurminsky suggest that X and autosomes may differ in their abilities to bind activator versus repressor proteins. We would modify their model by incorporating dosage compensation. Dosage compensation, the mechanism by which the expression of X-linked genes is reduced in XX females or increased in XY males, takes a long time to evolve to completion [11]. Thus, tissue-specific expression of X-linked genes would have to evolve without the benefit of full dosage compensation, and dosage inequality of X-linked genes that start to evolve toward tissue specificity would have to be tolerated until dosage compensation is completed. Autosomal genes without this extra hurdle may evolve tissue-specificity more readily.

The remaining issue is the evolution of genes that show specificity in male reproductive tissues. The autosomal bias of genes expressed specifically in male reproductive tissues may be explained by sexual antagonism (according to Scheme II of Figure 1) or by dosage compensation (according to Scheme III). Both schemes predict autosomal bias in this category of genes.

Mikhaylova and Nurminsky suggested that male reproductive tissues should belong in the same category as non-reproductive tissues (Scheme III). However, two ancillary observations suggest that male and female reproductive tissues may be driven in opposite directions by the same force, and that the driving force in the non-reproductive tissues is in a separate category. First, one may not expect dosage compensation in testis and the authors did notice that the key component of dosage compensation, msl-2, does not appear to function in testis. Second, genes specifically expressed in the accessory gland, which is strictly male reproductive, show a stronger autosomal bias than those specifically expressed in non-germline tissues (see Figure 4 of [6]), suggesting that the two male specific tissues, distinct from the non-sexual tissues, appear to behave similarly.

In conclusion, we suggest that two separate mechanisms, sexual antagonism and improper regulation of tissue-specificity for X-linked genes (perhaps connected to dosage compensation), drive the chromosomal distribution bias of ovary- and somatic tissue-specific genes, respectively. The chromosomal bias in male reproductive tissue-specific genes could be driven by either mechanism. Future work to clarify this issue will be important to our understanding of the structure, function and evolution of sex chromosomes.
